# Scaffold strategies combined with mesenchymal stem cells in vaginal construction: a review

**DOI:** 10.1186/s13619-021-00088-2

**Published:** 2021-08-02

**Authors:** Nicole Andréa Corbellini Henckes, Dalana Faleiro, Laura Chao Chuang, Elizabeth Obino Cirne-Lima

**Affiliations:** 1 Programa de Pós-Graduação em Ciências da Saúde—Ginecologia e Obstetrícia, Universidade Federal do Rio Grande do Sul (UFRGS), Porto Alegre, Brazil; 2 Laboratório de Embriologia e Diferenciação Celular, Centro de Pesquisa Experimental, Hospital de Clínicas de Porto Alegre, Porto Alegre, Brazil; 3 Departamento de Patologia Clínica Veterinária, Faculdade de Veterinária, Universidade Federal do Rio Grande do Sul (UFRGS), Porto Alegre, Brazil

**Keywords:** Mesenchymal stem cells, Scaffolds, Tissue engineering, Vaginal reconstruction

## Abstract

Tissue engineering has provided new treatment alternatives for tissue reconstruction. Advances in the tissue engineering field have resulted in mechanical support and biological substitutes to restore, maintain or improve tissue/organs structures and functions. The application of tissue engineering technology in the vaginal reconstruction treatment can not only provide mechanical requirements, but also offer tissue repairing as an alternative to traditional approaches. In this review, we discuss recent advances in cell-based therapy in combination with scaffolds strategies that can potentially be adopted for gynaecological transplantation.

## Background

Tissue engineering advances and cell-based therapies have presented promising opportunities for repairing tissue and organ defects as well as acceleration of the regenerative process. Given that the body has a limited self-regeneration ability of tissues and organs (Chen and Liu, 2016), the development of tissue engineering can offer an alternative tailored to meet the specific cases where vaginal reconstruction is required and also provide biological substitutes to restore or maintain tissue/organs function in order to improve the quality of life (Jakubowska et al., 2020). While there is no standard procedure for surgical repair, there are many surgical techniques that require multiple surgeries and often additional tissues and nonsurgical techniques (Schenke-Layland and Brucker, 2015; Unger and Paraiso, 2015). For women that present complex reproductive structural anomalies, several new techniques and ideas from different fields are leading to support surgical innovations.

In recent times, the therapeutic application of mesenchymal stem cells (MSCs) have been investigated and the outcome brings new expectations about the management and possible long-term effects in a wide array of disease models (e.g. vaginal agenesis) (Galipeau and Sensébé, 2018). The usage of MSC in combination with scaffolds is promising as a tool in the treatment of damaged tissues that have specific functions, once they create a favourable regenerative microenvironment (De Francesco 2019; Yi et al., 2017) even in cases where the patient has limited amount of available native organ tissue and when tissue regeneration is required.

A positive biological response in this interesting strategy includes the support for cell growth (e.g. scaffold matrices) and biological signals that guide secretory products, including immunoregulatory cytokines, growth factors and exosomes into the desired tissue (Klimek and Ginalska, 2020; Pittenger et al., 2019; Cherian et al., 2020). This aspect of the cell behaviour, combined with biomaterials, results in the release of different factors into the surrounding environment (Yi et al., 2017) leading the way for successful tissue regeneration (Reddy et al., 2020).

Our group has recently developed a PLGA/PIepox scaffold and the in vitro model MSCs have shown biological features in the proliferation ability in poly (lactic-co-glycolic acid)/ epoxidized poly (isoprene) (PLGA/PIepox) scaffold (Henckes et al., 2019; Guerra et al., 2018). As a result, many efforts have been focusing in the application of technologies involving an in vivo model approach to further extend the use to clinical practice as well as to restore or repair reproductive organs and other organs with similar tissue structures therapies.

This review aims to present the current knowledge acquired in our research group to contextualise and a perspective on the most important characteristic involving mesenchymal stem cells combined with scaffolds for tissue engineering in gynaecological application. The list of bibliographic material contains the relevant scientific literature on the subject and the analysis of the same was used to provide an overview of the combined use of mesenchymal stem cells and PLGA/PIepox.

## Main Text

### Vaginal agenesis requiring regenerative therapies

Scientific researchers have recently expanded their research involving stem cells to offer the opportunity to treat gynecological pathologies such as pelvic floor prolapse and uterine and vaginal reconstruction. New techniques and ideas from different fields are leading to surgical procedures innovations and these available therapies involve biological substitutes that can provide a favourable microenvironment for cells and tissues to grow and restore biological activities (Magalhaes et al., 2020). In this vein, considering the advances in scientific experiments, the tissue engineering may offer new therapies for vaginal reconstruction as well as congenital agenesis by combining cell therapy and new technologies to create new tissue.

When it comes to the condition of vaginal agenesis, it is important to notice that this gynecological pathology is linked to a complex anomaly involving reproductive structural formation problems it can occur in different situations with total or partial absence of the vagina (De Souza et al., 2012; Thomas and Brock, 2007).

There are some cases, such as patients with Mayer-Rokitansky-Küster-Hauser syndrome (MRKH) and transsexual individuals who desire a male-to-female sex reassignment, where the individuals are extremely affected by the absence of vagina. In both cases it is necessary to intervene with a reconstructive surgery to adapt the anatomy to a natural female appearance (Morais and Cortes, 2020; Dreher et al., 2018).

The principle is to surgically create a cavity for the vagina (Tarry et al., 1986) and submit patients to a series of surgical procedures combined that later require a continuous usage of vaginal dilators in order to keep the newly constructed physical structure, bring functionality and support sexual neo-organ epithelialization (Callens et al., 2014; Baptista  et al., 2016).

Thus, the current therapy is the uncomfortable, long and invasive process, briefly described in Table 1, not to mention that the surgery has to be performed twice in about 40% of the cases (Grimbizis et al., 2015; Oelschlager and Debiec, 2019).

**Table 1:** A short description about causes and current therapy of the vaginal reconstruction

Factors which can promote the vaginal abnormalities	Conventional therapies to vaginal reconstruction	Critical issues regarding conventional therapies
I. Genetic alterations	McIndoe technique: reconstruction of the vaginal canal through full-thickness skin grafting; surgical methods.	Continuous use of molds until complete epithelialization
II. Hormonal alterations	Frank’s technique: progressive dilation for distension and the creation of a vaginal neocavity; non-surgical methods.	Requires great motivation and persistence from patients
III. Epigenetic factors	Vecchietti technique; laparoscopic approach	Pain during vaginal traction, lack of lubrication and prolonged use of vaginal prostheses.

Therefore, surgical reconstructive approaches to replace the tissues with functionally equivalents would improve the outcome of reconstructive surgery and the quality of life compared to the currently available options (Ko et al., 2013; Rothberg and Atala, 2018). The chosen treatment will depend on the experience of the surgeon, considering that repeated surgeries might become more challenging with less successful outcomes over time (Unger and Paraiso, 2015).

Toward this goal, tissue engineering combining cells and scaffolds emerged as an alternative method for gynecological reconstruction and for that the selection of an appropriate scaffold is essential to provide tissue functionality providing an adequate anatomy (Sartoneva et al., 2018; Papadopulos et al., 2017; Wu et al., 2020), hence the importance of the approach involving PLGA/PIepox plus MSCs. Although this combination does not confer structural strength, it brings functionality and coating and must be combined with dilators in order to confer physical structure. Recently studies described the current belief is that biological materials used in vaginal construction are expected to provide a protective layer and allow tissue to undergo epithelization (Dias et al., 2020).

Considering the procedures offered to patients with gynecological pathologies, it is necessary to deeply understand the biological mechanisms involved in technologies that combine scaffolds and MSCs in order to provide better solutions to patients who require tissue reconstruction.

Several studies in the literature have already demonstrated results regarding the applicability of new scaffolds in tissue engineering for gynaecological reconstruction and treatments (De Filippo et al., 2003; De Philippo et al., 2008; Orabi et al., 2017; Boennelycke et al., 2011) (Table 2). Although they are biocompatible and appear as candidates in gynaecological application as describe in Table 2, there are no publications involving mesenchymal stem cells for this purpose so far.

**Table 2:** Main researches developed in in vivo experiments upon the potential of cell co-cultured in scaffolds to vaginal abnormalities

Reference	Study model	Regeneration strategy	Cells seeded on scaffolds	Benefits
De Filippo et al., 2003	Mice	Engineering vaginal tissues	Vaginal epithelial and smooth muscle cells + PGA	Neovascularization
De Philippo et al., 2008	Rabbit	Vaginal replacement	Vaginal epithelial cells and smooth muscle cells + PGA	Neovascularization and appropriate physiological responses
Raya-Rivera et al., 2014	Human	Tissue engineered autologous vaginal organs in MRKH syndrome + SIS	Epithelial and smooth muscle cells + SIS	Organized vaginal histology
Zhu et al., 2013	Human	MRKH syndrome	No cells; acellular dermal matrix	No complications; anatomic success 100% and normal sexual function
Panici et al.*,* 2015	Human	Canal lining in patients with MRKH syndrome	Mucosal vaginal cells; no scaffold	Normal and satisfying sexual intercourse

Further studies have shown the outcomes from a vaginal reconstruction using tissue-engineering biomaterial graft and reveals great results upon vaginoplasty and confirms the safety and effective procedure provide near normal sexual function (Zhu et al., 2013). In a new discovery involving autologous in vitro cultured vaginal tissue for vaginoplasty, Panicci et al. has highlighted that although there are several suggestions in different approaches altering the scaffolds and the biological combination (Table 2), there is not a consensus on what material should be used for the neovagina canal wall lining (Panici et al., 2015).

These promising results might bring innovative solutions. In this vein, considering the advances in experiments (Henckes et al., 2019; Guerra et al., 2020; Guerra et al., 2018), the option of tissue engineering with the combination of mesenchymal stem cells and scaffolds for patients who require additional tissues and need immediate and multiple reconstructive surgeries has increased considerably. Certainly, the use of seeded cells-therapy combined with scaffolds for vaginal reconstruction and others abnormalities is promising and requires further investigation.

### Biological function of MSCs in in vitro and in vivo model

**Fig. 1:**
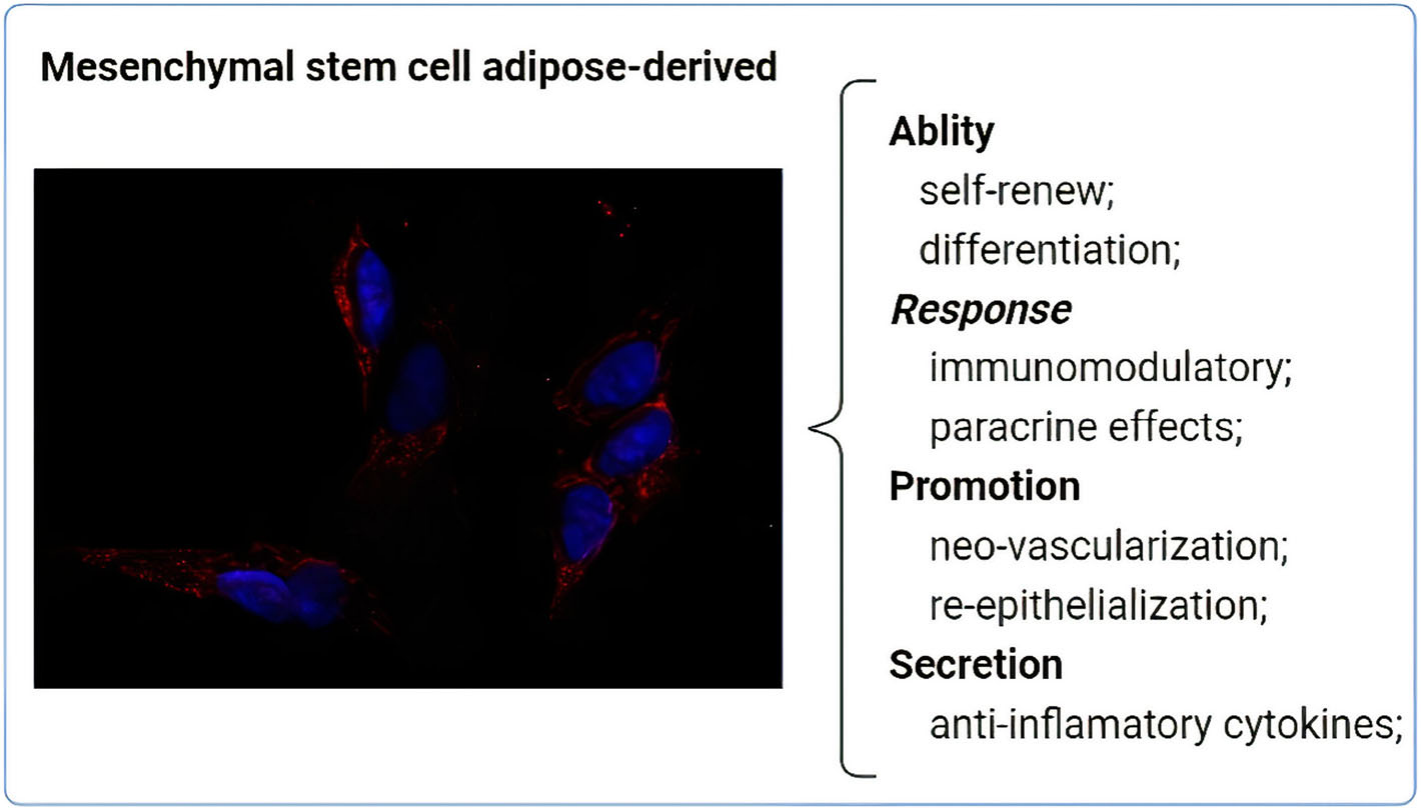
Brief description of mesenchymal stem cells (MSCs) functions. MSCs has self-renewal ability and differentiation potential in different lineages (e.g. adipocytes, chondrocytes and osteocytes) and can be isolated from different sources. MSC has immunomodulatory function acting by paracrine effect and is able to promote tissue neo-vascularize and re-epithelize as well secretes anti-inflammatory cytokines during tissue restoration

Mesenchymal stem cells have been extensively studied due to features such as self-renewal and differentiation properties, facility to isolate from tissues and manipulate that brings less ethical concerns and also a high in vitro expansion capacity (AghebatI-Maleki et al., 2019; Samsonraj et al., 2017). Beyond these features, MSCs have shown extraordinary results due to the ability to exhibit anti-inflammatory effects involving cytokines production and immunomodulatory activities as well as production of growth factors and capacity to migrate to damaged tissue (Guadix et al., 2017; Regmi et al., 2019; Gnecchi et al., 2008). Once MSCs are isolated, they must adhere to the plastic and be able of differentiate into osteocytes, chondrocytes and adipocytes lineages in in vitro culture under certain conditions (Saeedi et al., 2019; Luck et al., 2020) (Fig. 1).

In order to improve and maintain desired biological function and maximize the therapeutic effects produced by MSC it is necessary to prepare and optimize the in vitro culture. This strategy plays an important role in the MSC function and contributes to the efficacy of transplantation to the host tissue (Thirumala et al., 2013; Sart et al., 2014; Hu and Li, 2018). Consequently, all strategies involving tissue engineering that address the combination of cells and scaffolds should be tested in in vitro models under different controlled conditions in order to demonstrate the efficacy of the approach (Conci et al., 2020).

According to the review carried out by Uder et al. due to the ability of in vitro expansion, proliferation and self-renewal of MSCs, clinical usage has become attractive since it requires a number of cells far higher than those originally obtained from a donor sample, and - even after extensive expansion and manipulation in vitro - MSCs have shown the ability to maintain their function and performance (Uder et al., 2018).

Gowen et al. brought to their review the ideas that initially the MSC-based therapy was promising due to its ability to migrate in the target host tissue. Over the years, however, the ability of cells to secrete factors has been added as biological activity and linked to several beneficial effects (Gowen et al., 2020). Charras et al. supports the idea that the adequate MSC biological activity of the cell migration is a fundamental ability to the self-regeneration and depends on the protein expression and signalling (Charras and Sahai, 2014).

Over the years, based on studies that have been carried out highlighting the potential benefit of using MSC in cell-based therapy for organ and tissue reconstruction, new studies have been conducted with different types of MSCs as adipose–derived mesenchymal stem cells (ADSCs), which may have greater regenerative potential than other types of MSC such as Bone Marrow-Derived Mesenchymal Stem Cells (BM-MSCs) and MSCs derived from dental pulp tissues (DPSCs) (El-badri 2016; Luck et al., 2020; Forsberg et al., 2020). Although different types of MSCs share common stem cell properties, they differ regarding their population number, proliferation rates, differentiation abilities, and clinical outcomes (Mazini et al., 2019). Here we present some differences between the main characteristics of mesenchymal stem cells types.

The multipotent cells group of type BM-MSCs are present in the bone marrow stroma and capable of differentiating into several cell lines of the mesodermal and non-mesodermal cell types (Berebichez-fridman, 2018). They are linked to the maintenance of a microenvironment based on the secretion of chemokines and growth factors that contribute to cell proliferation, self-renewal and differentiation (Leuning et al., 2018). Furthermore, BM-MSCs express intermediate levels of major histocompatibility complex (MHC) class I molecules which contribute to immune tolerance due to the low immunogenicity and the immunosuppressive effect (Machado et al., 2013). These cells are capable of osteogenic, chondrogenic, adipogenic, neurogenic and cardiogenic differentiations. However, the use of BM-MSCs has some disadvantages, such as a low number of MSCs (0,01% a 0,001%) and the isolation depending on the patient status and the volume of aspirates (Bydlowski et al., 2009; Pontikoglou et al. 2011).

The adherent cells of type DPSCs have the morphology like fibroblasts and have been confirmed by their ability to differentiate into neural ectodermal cells and adipocytes, odontoblasts, osteoblasts, chondrocytes and myoblast cells of mesodermal origin, confirming their plasticity besides high proliferation capacity (Mattei et al., 2021). When compared to BM-MSCs, DPSCs have a greater potential ability to induce mineralization, but may be more restricted in their differentiation potency (Ma et al., 2019).

DPSCs cells are located within the dental crown, in a niche that houses the connective tissue. The resident tissue cells are a heterogeneous population represented by stromal fibroblasts and accompanied by vascular and inflammatory immune cells (Aydin and Şahin, 2019). DPSCs do not seem to express a marker that exclusively identifies them and might have an immunophenotype difference from that others MSCs types. It is possibly due to the presence of different subpopulations of MSCs in dental pulp that have different biological activities. Their limitations are the risk of contamination during collection and the limited number of cells initially available for therapy (Ledesma-Martínez et al., 2016; Huang et al., 2009; Kichenbrand et al., 2019).

The cells of type ADSCs can be maintained and expanded in culture for long periods of time without losing their differentiation capacity, leading to abundant quantities and with a high cellular activity being increasingly used for cell therapy purposes (Sterodimas et al., 2010). MSCs obtained from adipose tissue is the main stem cell source due to its accessibility and abundance when compared to other sources. Their frequency is about 2% in its stromal vascular fraction and it is the highest one when comparing all tissues (Ntege et al., 2020; Mazini et al., 2019). ADSCs also maintain their potential to differentiate into cells of mesodermal origin and are commonly known for their low immunogenicity, modulatory and paracrine effects (Mazini et al., 2019). ADSCs approaches seem to be safer and more efficient in terms of the side effects reported.

The development of allogeneic approach means that ADSCs can be isolated from a volunteer donor, expanded and cryopreserved to supply the need for tissue repair. Thus, cells that were obtained from a single donor can be used to treat different patients due to an immune-privileged characteristic (Wang et al., 2020). ADSCs secrete higher amounts of pro-angiogenic molecules, such as extracellular matrix components and metalloproteinases (MMPs) and vascular endothelial growth factor (VEGF) compared with other MSC. This suggests that ADMSC may be preferred over other MSC populations for augmenting therapeutic approaches dependent upon angiogenesis (Mazini et al., 2019; Costa et al., 2021).

The scientific researchers have focused on the use of ADSCs due to its easy of obtaining and expansion process along with regenerative potential. In addition, ADSCs can assist in the repair of damaged tissue through cytokines secretion and growth factors from the paracrine and immunomodulatory effects. As expected, there are numerous advantages of using ADSCs in cell-therapy owing to its biocompatibility and biological characteristics (Conci et al., 2020).

Considering the promising preliminary clinical translation to the in vivo model, the importance of understanding the tissue repair process needs to be highlighted. MSC-specific tissue reconstruction mechanism occurs when MSCs prepare a microenvironment and the enzymes present lead them towards the specific organs and tissues. Once the MSCs have reached the specific target, the cytokine releasing process begins in response to the inflammatory stimulus (Naji et al., 2019; Madrigal et al. 2014).

Contributing to this process, there are other remarkable properties involved in MSC cell-therapy that also play important roles in the treatment efficacy - besides signalling molecules, they also connect tissue, influence the therapeutic response, contribute to immunomodulatory activities and mediate and regulate angiogenesis and apoptosis processes (Wang et al., 2018; Langhans 2018). Considering these characteristics and all the benefits, the use of MSCs can be seen as vital for tissue reconstruction strategies.

### PLGA/PIepox scaffold in tissue reconstruction

The majority of the current existing strategies for tissue reconstruction involve the development of new scaffolds (Lanza et al., 2020). Scaffolds plus cells approaches became an emerged field to be filled with new materials such as PLGA/PIepox in cases where there is a need for tissue replacement/restoration.

A wide number of scaffolds combined with cells have been presented as a viable option for vaginal reconstruction and for repair genital and gynaecological structures (Laurence et al., 2015). Since both the chemical characteristics and versatility are the main advantage of synthetic blends (Almouemen et al., 2019), we can consider that PLGA/PIepox stands out as the more suitable approach.

Many different polymeric scaffolds have been developed in both scientific researches and in clinical tests and mainly differ in comparison to the compounds. As described in Table 3, these scaffolds include in their composition: collagen (Dong and Lv, 2016), alginate (Bhattarai et al., 2006), polyglycolic acid (PGA) (De Filippo et al., 2003; Bissoli and Bruschini, 2018), poly lactic-co-glycolic acid (PLGA), poly(L-lactic acid) (PLLA) (Kuo et al., 2010; Yang et al., 2004) and poly(ε-caprolactone) (PCL) (Zhang et al., 2016).

**Table 3:** Possibilities of applications of some scaffolds available as well as advantages and perspectives

Material	Application perspective	Advantage
**Alginate**	→Skin, →Cartilage, →Bone, →Liver, →Cardiac tissue.	→Biocompatibility, →Fast degradation, →Biodegrability.
**Collagen**	→Nerve, →Bone, →Cartilage, →Tendon, →Ligament, →Blood vessel, →Skin.	→Low immunogenicity, →Good permeability, →Biocompatibility, →Biodegradability.
**PGA**	→Vaginal reconstruction, →Pelvic floor repair.	→Hydrophilic, →Biodegradability, →Non-toxic, →Biocompatibility.
**PLGA**	- Intestine, - Liver.	- Biodegradability, - Suitable mechanical properties.
**PLLA**	→Ligament tears, →Central nerve system.	→Biodegradability, →Resorbable.
**PCL**	- Meniscus.	- Biodegradability.
**PLGA/PI (Cellprene®)**	→Cranioplasty, →Pneumology.	- Resorbable, - Biocompatibility.
**PLGA/PIepox**	→Tissue reconstruction, →Biological dressing.	→Hydrophilic, →Resorbable, →Biocompatibility, →Non-cytotoxic, →Suitable mechanical properties, →Easily fabricated, →Low cost.

Among those potential applications, one of the most promising uses is for developing fibrous scaffolds that can mimic the physical structure and provide an ideal environment to promote cell growth (Atala 2011; Liu et al., 2013). Another possibility, from a tissue reconstruction perspective, is to combine or join different types of scaffolds in an attempt to maximize their advantages (Conci et al., 2020).

One of the potential applications of PLGA/PIepox is a combination between PLGA, that shows a biocompatible characteristic, and Poly (isoprene) that exhibits strong angiogenic properties (Guerra et al., 2018; Kerche-Silva et al., 2018). The epoxidation (epox) procedure is considered one of the most important processes in organic synthesis due to its particular characteristic of increasing the hydrophilicity of rubber (Guerra et al., 2018). These characteristics are important for cells to adhere in greater quantity and to promote their fixation in the host tissue, so the choice of scaffold is fundamental for the success of the regenerative therapies.

Regardless of the fabrication approach used, scaffolds have a key role in the integration in new tissue having a crucial performance in the host microenvironment (Dias et al., 2020), although the barrier to scaffold translation demands specific and appropriated conditions to successfully reconstruct tissue and organs defects (Naderi et al., 2020).

Previously, a study involving PLGA/PI blend (known commercially as Cellprene®), described the production process, the physical and chemical characteristics and the in vivo application in an animal model scaffold usage. This publication reports the efficacy of manufactured PLGA/PI fibres suggesting that this blend can be an attractive alternative for tissue engineering (Marques et al., 2016). In parallel, a study concerning about a new application of PLGA/PI as a stent was developed and the material implanted into the trachea of rabbits (Schopf et al., 2018). Schopf’s study demonstrated the occurrence of an inflammatory reaction in the adjacent tissue to PLGA/PI polymeric implanted fragment (Schopf et al., 2018). In a recent progress, novel studies were conducted to improve and overcome limitations in the process of PLGA/PI (Cellprene®) fabrication as well as its chemical composition where modifications have been applied and new applications were tested, resulting in a new scaffold epoxidized called PLGA/PIepox (Guerra et al., 2018; Henckes et al., 2019).

With the new compound, additional advantages were obtained as a consequence of its characteristics. When the polymer PLGA/PIepox was compared to Cellprene® in biostudies, it was possible to demonstrate higher performance to the epoxidized polymer, suggesting a positive clinical application perspective. For this, our research group is developing a relevant study to the overall in vivo assessment of tissue compatibility and biological evaluation of the PLGA/PIepox produced by electrospinning. Considering the results obtained so far by our research group on the effect of ADSC (Martins et al., 2019; Vidor et al., 2018; Beheregaray et al., 2017), there is an indication that they are a great candidate for the experimental tests carried out in an in vivo model.

### Potential applications of PLGA/PIepox and MSC-based cell therapy

In recent years, cell-based therapy has surfaced as a promising therapeutic approach and has many enthusiastic researchers that consider it an opportunity to restore tissues and organs (Golchin et al., 2020). Regarding the safety and efficacy of MSCs therapies, it is important to consider that MSCs do not express major histocompatibility complex (MHC) antigens which are involved in the antigen recognition by the immune system (Rawat et al., 2019; Cherian et al., 2020; Lukomska et al., 2019). For this reason, MSCs are not recognized as foreign cells giving them the ability not to induce rejection reactions when transplanted to different individuals or species. This particular aspect of MSCs has become commercially attractive and clinically practical usage for cell therapy, since it enriches the repair potential from cell transplantation and promotes restore function (Cherian et al., 2020; Lee et al., 2020). Scarritt, et al. interestingly indicates that scaffolds can increase and improve the MSC differentiation and this probably occurs due to the interaction with tissue host (Scarritt et al., 2015).

**Fig. 2:**
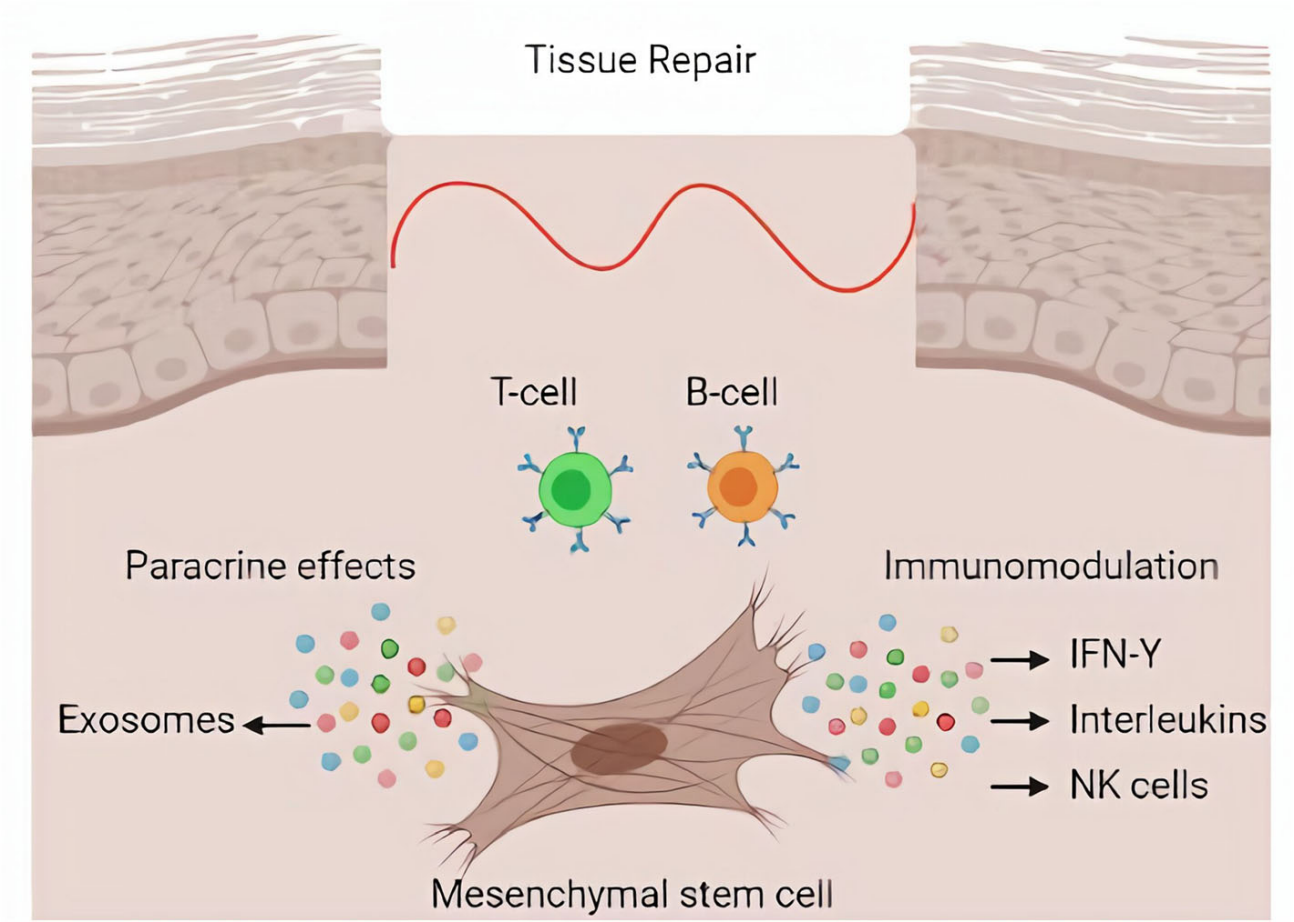
Representative image of the mechanism of action of cells in the tissue repair shows the immune response of MSCs by immunomodulatory secretion factors and the paracrine effect of MSCs through secretion of exosomes which release of the biological active content for immunomodulatory effect

Current cell-therapy approaches, mainly using MSC, can greatly impact the regenerative medicine, as they have a capacity to migrate into damage tissue and to release paracrine factors, which are able to decrease inflammation and promote immunomodulation producing a potential anti-apoptotic benefit by cytokines production and secretion (Fig. 2) (Fu et al., 2019; Brown et al., 2019; Hong et al., 2019). Despite being one of the main tools used today in cell therapy, it is still important to elucidate the mechanisms through which they interact with the host tissue. Hence, novel strategies for exploring the biological activities would help us to make better choosing your approach for cell-therapy (Li et al., 2019).

Alternatively, cell-therapy approaches combined with several scaffolds have been considered to tissue reconstruction and the biocompatibility of the scaffold is a fundamental feature to the successful engineering of tissues in regenerative medicine (Yesmin et al., 2017). Considering the promising therapeutic approach, it is important to understand the mechanisms of MSC interactions with scaffolds since they can provide structural property and also improve cells delivery in the host tissue and maximize their beneficial potential either long-term or short-term (Lee et al., 2020; Zonari et al., 2015; Khaled et al., 2011). To address these challenges, researchers are improving the composition of scaffolds and performing necessary tests to make them available for clinical practice (García-Gareta et al., 2013).

**Fig. 3:**
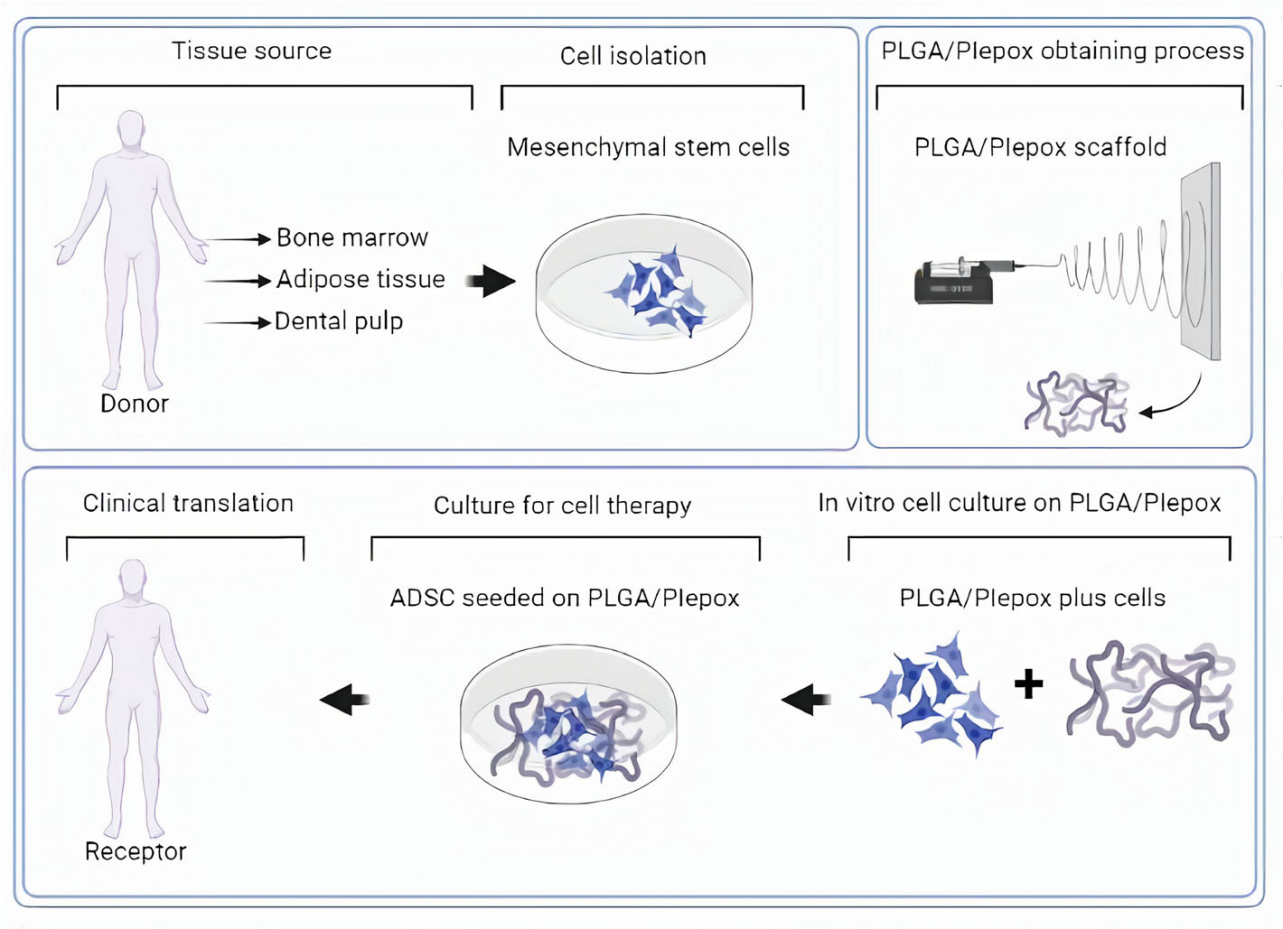
Schematic process involving MSC-therapy combined with PLGA/PIepox. The tissue sample is obtained from a donor. The cells are isolated, characterized and expanded in culture. In parallel to this, the process of obtaining the PLGA/PIepox scaffold is initiated by electrospinning technique. After obtaining of the scaffold and characterizing the cells, these cells are attached to a scaffold. After adding the cells to the PLGA/PIepox scaffold, the construction is implanted into the host

Therapies combining stem cells and scaffolds appear to be a new attempt to treat damaged tissues and organs (Fig. 3). The clinical translation importance of MSCs, especially when combined with scaffolds, in recent times is focused on off-the-shelf cells (Thirumala et al., 2013). However, although there are advances involving cell-based therapy and scaffolds so far, there is a lack of treatment focusing on gynecological approaches with the purpose of accelerate the tissue repairing process and to promote regeneration of damaged or lost tissue by the ADSC and scaffolds regenerative properties. Therefore, a promising new therapeutic opportunity could be explored (De Coppi 2013; Wu et al., 2012). According to Garcia-Garreta et al. the potential beneficial effects of ADSC integration with scaffolds in clinical application to restore the biological activity of the host tissue plays a key role in tissue engineering (García-gareta et al., 2020).

Although there are strategies to the translation in cell-therapy, these technologies are still far from the clinical practice. In attempt to bring it as a novel alternative to conventional therapies, it’s necessary to overcome certain challenges, such as defining a cell source that could be used reliably and the ideal scaffold to provide an optimal environment for potential tissue regeneration (Yang et al., 2020).

According to Thirumala et al. it is important to mention that depending on the complexity involving the lack of tissue and organs this approach can take many years to finalize, due to the evaluation of the cell-based therapy and scaffolds having to be proven in the long-term (Thirumala et al., 2013). Bouten et al. highlights that, to address these challenges, understanding and acknowledging the complexity of the processes involving stem cells and scaffolds in biological responses in tissue regeneration is important (Bouten et al., 2012).

Within this challenging perspective, scientific researchers need to find an alternative to overcome these disadvantages and make the engagement of these new therapies with innovative potential and great therapeutic promise more plausible and attractive.

## Conclusions and future perspectives

The aim of regenerative medicine applied to vaginal tissue engineering is to overcome the main difficulties encountered with conventional approaches. To overcome the side effects of the standard treatments, a combination of PLGA/PIepox plus MSCs might emerge as a promising technique into biomaterials combined with cell-based therapy. We have been developing this combination to be capable of interacting with tissue host and promote tissue remodelling. This potential offers hope to patients with diseases that are often ignored or treated inadequately with non-effective treatments. PLGA/PIepox combined with MSCs may be consider as a consistent opportunity and affording advantages abound for the clinical application and can be a key challenge to patients which requiring extensive vaginal reconstruction surgery. Recent progress suggests that cell-based tissue engineering strategies in female organs may have a great potential for regenerative medicine that would benefit of tissue replacement or repair (Atala 2012; Sadri-Ardekani and Atala, 2015; Shea et al., 2014; Schenke-Layland and Brucker, 2015). Yet, it is still necessary to evolve in studies with the usage of signalling, such as synthetic factors or isolated cell factors in attempt to increase the treatment biosafety. In addition, in another perspective, an evolution in the use of combined scaffolds is suggested in order to provide mechanical structure and *stimuli* for a more adequate formation of the neovagina, surpassing the chemical characteristics of the current scaffolds.

## Data Availability

Not applicable.
